# Frailty and Sarcopenia in Elderly Diabetes Mellitus

**DOI:** 10.3390/jcm15103706

**Published:** 2026-05-12

**Authors:** Ashish Puskur, Poonam Ashok Kamath, H. Manjunatha Hande, Shreesha Nagaraju, Vijayendra Kedage

**Affiliations:** 1 Department of General Medicine, Kasturba Medical College, Manipal Academy of Higher Education, Manipal 576104, India; ashish.puskur@manipal.edu (A.P.); shreesha.n@manipal.edu (S.N.); 2 Department of General Surgery, Kasturba Medical College, Manipal Academy of Higher Education, Manipal 576104, India; vijayendra.kedage@manipal.edu

**Keywords:** frail, sarcopenic, elderly diabetic, Edmonton Frailty score, SARC F index, glycemic control

## Abstract

**Background:** With the global rise in the ageing population and type 2 diabetes mellitus (T2DM), elderly individuals are increasingly prone to complications like sarcopenia and frailty, which are conditions linked to muscle loss and functional decline. These syndromes contribute significantly to morbidity and healthcare burden but remain underestimated in older adults with diabetes. Indian data regarding their prevalence and correlation with glycemic control is limited. **Materials and Methods:** This hospital-based observational study was conducted over 22 months at Kasturba Medical College, Manipal, involving 160 diabetic patients aged ≥60 years. Clinical features, comorbidities, and diabetes-related complications were recorded. Frailty was assessed using the Edmonton Frailty Scale (EFS), and sarcopenia risk was evaluated using the SARC-F questionnaire. Descriptive statistics and Spearman’s correlation were used for analysis. **Results:** The mean age of participants was 69.98 ± 5.86 years, with 63.1% being male. Fatigue was the most common clinical presentation at 10.6%, while cardiovascular disease was the leading complication at 28.1%. Frailty or pre-frailty was identified in 35% of the population, and 36.9% were at risk for sarcopenia. A strong correlation was found between frailty and sarcopenia risk (*p* < 0.001). **Conclusions:** Frailty and sarcopenia are common and closely linked in elderly diabetes, though not significantly associated with glycemic control as both hypoglycemia and hyperglycemia could contribute to frailty.

## 1. Introduction

Diabetes Mellitus is thought to impact around 828 million adults globally, with a worldwide age-standardised prevalence of about 14 per cent among the adult population [1].

Compared with individuals in all other age groups, adults aged ≥65 years have the highest prevalence of diabetes (29.2 versus 4.8 per cent among those ages 18 to 44 and 18.9 per cent among those 45 to 64 years). In most developed countries, a chronological age of 65 years is considered the definition of elderly, but in many developing countries, including India, the accepted cutoff is 60 years [2]. As people age, the likelihood of developing type 2 diabetes rises, primarily due to a decline in insulin production and a rise in insulin resistance. These changes are frequently influenced by factors such as obesity and muscle loss [3,4]. In older adults, changes in diet and lower physical activity can lead to decreased lean body mass and increased adiposity, particularly visceral adiposity, contributing to insulin resistance [3]. The decrease in muscle bulk, strength, and function linked to diabetes contributes to sarcopenia, frailty, and eventual disability, with complications and other existing health issues related to diabetes being partially responsible for the decline in physical function [5].

Frailty results from the combination of ageing and certain chronic illnesses and conditions like diabetes that impair functional systems and ultimately lead to sarcopenia. Much of the clinical presentation of frailty can be attributed to sarcopenia, which is strongly linked to decreased physical performance [6]. India, recognized for having the second-largest elderly population in the world, features a distinctive and intricate social framework that reflects its diverse cultural, economic, and historical influences. These factors contribute to unique challenges in the ageing population within the broader societal context. Frailty can be targeted for treatment; therefore, early identification of frailty is important [7]. Previous studies have demonstrated varying prevalence of sarcopenia and frailty among elderly diabetic patients [8,9]. Frailty is increasingly recognized as a key complication of diabetes in older adults, contributing to adverse outcomes such as disability, hospitalization and mortality [10]. Sarcopenia has been redefined by the European Working Group on Sarcopenia in Older People (EWGSOP2), emphasizing muscle strength as a key determinant [11]. Frailty and sarcopenia have a profound effect on functional independence and quality of life, yet their relationship with glycemic control in the Indian elderly diabetic population is still not well understood. Understanding these relationships may help improve individualized diabetes management strategies for the development of more tailored and holistic strategies. By understanding these connections, healthcare providers may enhance metabolic outcomes and improve physical resilience in patients with diabetes. Individualized approaches could address the unique needs of each patient, ultimately leading to better management of the condition and improved quality of life.

While previous studies have assessed sarcopenia using AWGS criteria and objective muscle measurements, our study uniquely utilizes the SARC-F questionnaire alongside the Edmonton Frailty Scale. This approach allows rapid, clinically applicable screening in resource-limited settings. Furthermore, unlike prior studies, we specifically explore the relationship between frailty, sarcopenia risk and glycemic control in elderly Indian diabetic patients, highlighting their independence from HbA1C levels.

## 2. Materials and Methods

### 2.1. Study Design and Setting

We conducted a hospital-based cross-sectional observational study in the Department of General Medicine at Kasturba Medical College, Manipal, a tertiary care teaching hospital in Karnataka, India. The study was carried out over a duration of 22 months, from October 2023 to July 2025, with sample recruitment done from 12 November 2023, and involved elderly patients with type 2 diabetes mellitus (T2DM), both outpatients and inpatients, who presented for evaluation or treatment of diabetes complications or follow-up care. Each participant was assessed at a single time point; the study design is cross-sectional.

### 2.2. Study Objectives

The study was designed with the primary objective of evaluating elderly individuals with T2DM for their clinical features, comorbidities, and diabetes-related complications. Additionally, the prevalence of frailty and sarcopenia risk was assessed using the Edmonton Frailty Scale (EFS) and the SARC-F questionnaire, respectively. A secondary objective was to determine the correlation of frailty and risk of sarcopenia with glycemic control, as measured by glycated haemoglobin (HbA1c).

Additionally, the study aimed to explore the potential applicability of simple screening tools in routine clinical practice.

### 2.3. Inclusion and Exclusion Criteria

Participants were included in the study if they were aged 60 years or older, had a confirmed diagnosis of type 2 diabetes mellitus based on WORLD HEALTH ORGANIZATION (WHO) guidelines, and were willing to provide informed consent. Patients were excluded if they were bed-bound, had significant cognitive impairment or psychiatric illness that would interfere with assessments, or were experiencing an acute illness such as stroke, malignancy, or severe infection at the time of evaluation. These criteria ensured the inclusion of a stable population that could reliably undergo functional and cognitive assessments.

### 2.4. Sample Size and Sampling

The sample size was estimated based on an expected prevalence of frailty/sarcopenia in elderly diabetic patients derived from previous studies, assuming a prevalence of approximately 30–40%. Using a 95% confidence level and a 5% margin of error, the minimum required sample size was calculated to be approximately 150 participants. A total of 160 participants were included to account for potential variability and improve the robustness of estimates.

Given the cross-sectional observational design of the study, formal power calculation for hypothesis testing was limited; however, the chosen sample size is comparable to similar studies in the literature and is considered adequate to detect clinically meaningful associations.

### 2.5. Study Procedure

After obtaining informed consent, each participant underwent a structured clinical interview to collect detailed information regarding age, sex, duration of diabetes, treatment modality, and presence of comorbid conditions such as hypertension, dyslipidemia, cardiovascular disease, and others. Cardiovascular disease was defined as the presence of ischemic heart disease or heart failure as documented in patient medical records or based on prior physician diagnosis. A thorough physical examination was conducted, including measurement of weight, height, body mass index (BMI), and blood pressure. Based on BMI, individuals were classified as normal, overweight, obese class I and obese class II. The presence of diabetes-related complications was assessed systematically: neuropathy was evaluated through monofilament testing and vibration perception threshold; nephropathy was assessed using urine albumin-to-creatinine ratio and serum creatinine levels; and retinopathy was documented based on prior ophthalmologic evaluation or fundoscopy when available.

Frailty was evaluated using the Edmonton Frailty Scale (EFS), a validated multidimensional tool comprising 10 domains: cognition, general health status, functional independence, social support, medication use, nutrition, mood, continence, and functional performance via the Timed Up and Go test. The total score ranges from 0 to 17 and categorizes individuals as: not frail (0–4), vulnerable (5–6), mildly frail (7–8), moderately frail (9–10), and severely frail (≥11). Sarcopenia risk was screened using the SARC-F questionnaire, a validated and simple as well as a reliable screening tool for sarcopenia in both community and clinical settings [12]. This questionnaire includes five components: strength, assistance in walking, rising from a chair, climbing stairs, and a history of falls. Each item is scored from 0 to 2, and a total score of 4 or more suggests risk of sarcopenia. These tools were selected due to their simplicity, ease of administration, and prior validation in geriatric populations, including use in both community and clinical settings. The SARC-F questionnaire has been widely validated as a reliable screening tool for sarcopenia across diverse populations, while the Edmonton Frailty Scale has demonstrated good validity and reliability in assessing multidimensional frailty in older adults. Their applicability in resource-limited settings further supports their use in the present study [11,12]. Previous studies have also demonstrated the applicability of these tools in Asian and Indian populations, supporting their relevance in the current study setting [13]. Glycemic control was assessed by measuring HbA1c, using high-performance liquid chromatography (HPLC)-based standardised assays. HbA1c levels were compared with both frailty and sarcopenia scores. Body mass index was correlated with glycemic control, frailty and sarcopenia scores.

### 2.6. Statistical Analysis

Data collected from patient interviews and clinical assessments were entered into Microsoft Excel 2019 version 1808. and analysed using IBM SPSS Statistics version 27.0. Descriptive statistics were used to summarise demographic characteristics and prevalence rates. Continuous variables such as age, BMI, and HbA1c were expressed as mean ± standard deviation, and categorical variables such as gender, presence of frailty, and sarcopenia risk were expressed as frequencies and percentages. The relationship between HbA1c, frailty, and sarcopenia scores was assessed using Spearman’s rank correlation coefficient due to the non-parametric nature of the data. A *p*-value less than 0.05 was considered statistically significant. In addition to Spearman’s correlation, partial correlation analysis was performed to control for potential confounding variables such as age, BMI, and duration of diabetes. This allowed a more accurate assessment of the independent relationship between HbA1C, frailty and risk of sarcopenia. The statistical analysis in this study is primarily based on correlation methods, which provide insights into associations but do not establish causality. The cross-sectional design and absence of multivariable regression models limit the ability to fully adjust for confounding factors. The study is reported in accordance with STROBE guidelines.

## 3. Results

Baseline demographic and clinical characteristics are summarized in Table 1. The study population had a mean age of 69.98 ± 5.86 years, with a male predominance. The average BMI was 24.5 ± 4.1 kg/m^2^, and the mean duration of diabetes was 9.8 ± 6.4 years. Most participants (86.3%) were on oral hypoglycemic agents, and 20% required insulin therapy.

Hypertension was the most common comorbidity, followed by dyslipidemia and ischemic heart disease. Regarding clinical presentation, fatigue was the predominant symptom reported. Interestingly, 23.8% of the patients were asymptomatic and were evaluated during routine diabetic follow-up. About 51.9% had at least one documented diabetes-related complication. Cardiovascular disease was the most common complication. 7.5% had experienced a hospital admission in the past year due to hyperglycemia or hypoglycemia.

Frailty categories based on EFS (vulnerable, mild, moderate, severe) and sarcopenia risk were observed in a substantial proportion of participants. Among the SARC-F domains, difficulty in climbing stairs was reported most frequently (30%), followed by needing assistance for walking (24%) and difficulty in rising from a chair (18%).

**Figure 1:**
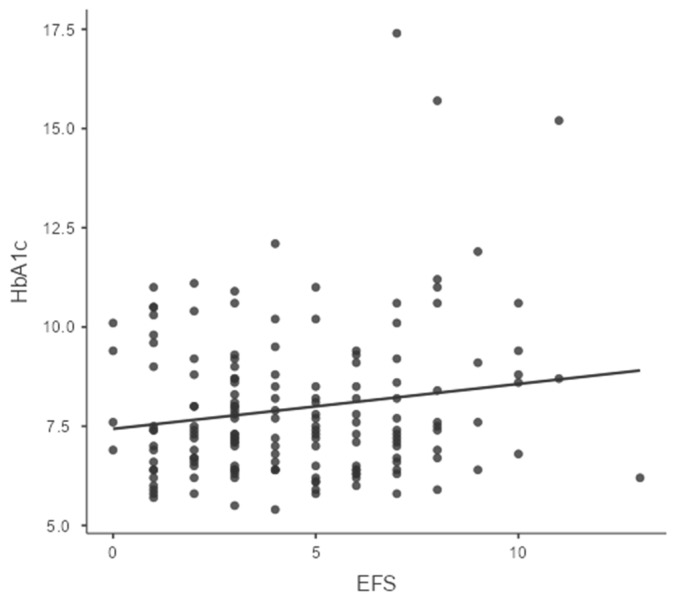
Correlation of EFS and HbA1c (*p* value 0.276).

**Figure 2:**
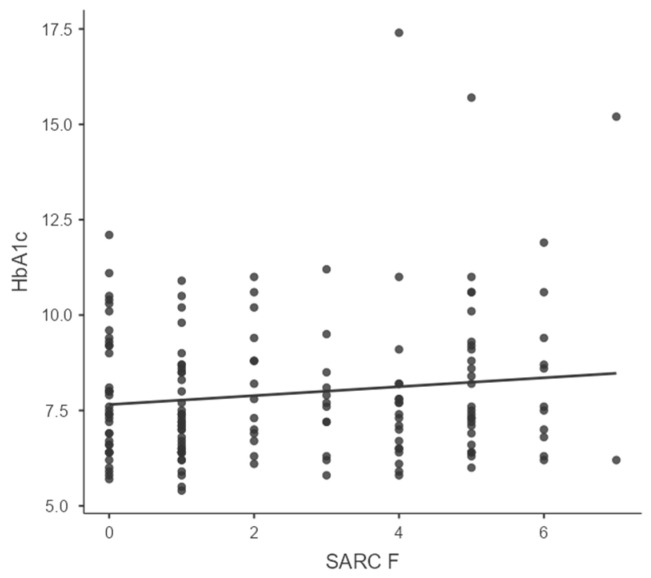
Correlation of SARC F and HbA1c.

With respect to glycemic control, the mean HbA1c of the cohort was 8.4 ± 1.4%, indicating suboptimal control in most patients. A majority (62.5%) had HbA1c levels above 7.5%, and only 18.8% had values below 7%, which is considered optimal. Poor glycemic control was more prevalent among individuals with a longer duration of diabetes, insulin use, and higher BMI. However, the correlation between glycemic control and frailty or sarcopenia risk was weak and statistically nonsignificant. Specifically, Spearman’s rho for HbA1c and EFS was 0.087 (*p* = 0.276), and for HbA1c and SARC-F was 0.065 (*p* = 0.411), as shown in Table 2 and Figure 1 and Figure 2. This implies that while frailty and sarcopenia are common in elderly diabetics, they may occur independently of short-term glycemic control.

**Figure 3:**
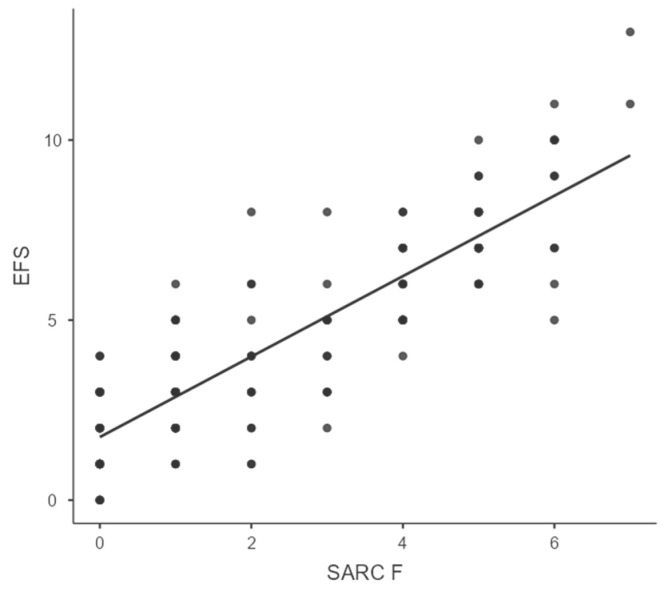
Correlation of SARC F and EFS.

A strong positive correlation was found between the EFS and SARC-F scores, with Spearman’s rho of 0.856 (*p* < 0.001), as shown in Table 2 and Figure 3, suggesting a substantial overlap between frailty and risk of sarcopenia in the study population. This reinforces the clinical interrelationship of these two syndromes in elderly diabetics.

BMI was found to have a significant association with glycemic control, where individuals with higher BMI demonstrated poorer control. However, no significant association was observed between BMI and either frailty or risk of sarcopenia. When comparing gender differences, males exhibited a slightly higher prevalence of sarcopenia risk (40.3%) compared to females (32.8%), whereas frailty was more frequently observed in females (18%) than in males (11.8%). Nonetheless, these gender-based differences were not statistically significant.

In summary, frailty and risk of sarcopenia were highly prevalent and closely interrelated among elderly individuals with type 2 diabetes, although no significant association was found between these conditions and glycemic control.

## 4. Discussion

### 4.1. Demographic Profile

The present study included 160 elderly adults with T2DM, with a mean age of 69.98 ± 5.86 years. The majority (55.6%) were in the 60–70 age group, indicating a predominance of early older adults. Males constituted 63.1% of the study population. This demographic distribution is comparable to the cohort described by Kolaric et al. (2022), which also demonstrated a male predominance and a similar age group distribution [13].

The observed demographic pattern may reflect healthcare-seeking behavior and gender based access to tertiary care services in the Indian context, where males are more likely to present for evaluation.

### 4.2. Clinical Features

The most commonly reported symptom was fatigue (10.6%), followed by polyuria/polydipsia and other nonspecific complaints. These symptoms are consistent with atypical presentation of diabetes in elderly individuals, where classic osmotic symptoms may be less prominent due to gradual metabolic adaptation and the coexistence of comorbidities. The relatively low prevalence of acute symptoms may also indicate chronic disease status with partial metabolic compensation, as described in prior literature on geriatric diabetes [4].

### 4.3. Complications

Among complications, cardiovascular disease (28.1%) was the most common, followed by neuropathy, nephropathy, retinopathy and peripheral artery disease. In comparison, Kolaric et al. (2022), which involved 382 individuals with type 2 diabetes mellitus, reported substantially higher rates of diabetic complications [13]. This discrepancy may be attributed to differences in study populations, as their cohort likely included a broader and possibly more advanced disease spectrum. Additionally, earlier diagnosis, improved glycemic management and regular follow-up in a tertiary care setting may have contributed to the relatively lower complication rates observed in our study.

### 4.4. Co-Morbidities

In the present hospital-based study, hypertension was the most prevalent co-morbidity among individuals with diabetes mellitus (78.1%), followed by dyslipidemia (20%), ischemic heart disease (19.4%), hypothyroidism (17.5%), and chronic kidney disease (9.4%). In contrast, the population-based review by Kiran SR et al. (2024) reported slightly higher prevalence rates for hypertension (82.1%), dyslipidemia (77.2%), and chronic kidney disease (24.1%). This discrepancy may indicate differences in population characteristics, healthcare access, and diagnostic practices between community-based settings and tertiary hospitals. Furthermore, while 79.5% of participants in our study were classified as overweight or obese, the review article reported a similar obesity rate of 78.2%. This highlights the high prevalence of obesity as a comorbidity in both clinical and community settings [14].

### 4.5. Treatment Patterns

Most patients (79.4%) were treated with oral antidiabetic drugs, with high reported compliance. Among these, metformin, DPP-4 inhibitors and sulfonylureas were the most used. Only 3.1% were on insulin alone, and 17.5% received combined OHA and insulin. This supports effective outpatient care. Notably, hypoglycemic episodes were predominantly observed in patients receiving insulin or sulfonylureas, which is consistent with their known pharmacological risk profiles. These findings highlight the importance of individualized therapy in elderly patients to minimize adverse effects while maintaining adequate glycemic control.

### 4.6. Frailty Proportion

In our study, we found that 35% of participants were classified as frail, 20.6% vulnerable, 9.4% mild frailty, 4.4% moderate, and 0.6% severe frailty. This is lower than the prevalence of frailty (67.3%) reported by Mange et al. (2021) [15], which may be explained by differences in mean age and study settings, as their cohort included older individuals with higher baseline vulnerability. However, our findings are in line with Indian data reported by Singhal et al. (2023), who found frailty in 42.34% and pre-frailty in 47.64% of elderly Indian adults, with a Karnataka-specific prevalence of 28.48% [7]. This suggests that regional and demographic factors significantly influence frailty prevalence. The presence of pre-frail population (20.6%) is clinically relevant, as this stage is potentially reversible with appropriate interventions.

### 4.7. Sarcopenia Proportion

The prevalence of sarcopenia risk (36.9%) is higher than that reported in multicountry analyses, such as S. Tyrovolas et al. [16] but closely aligns with Indian data from Rahman et al. [17]. This variation may be attributed to differences in diagnostic criteria and tools used. The use of SARC-F in our study emphasizes functional impairment rather than direct muscle mass measurement, which may identify individuals at earlier stages of decline. Additionally, ethnic differences in body composition and lifestyle factors such as physical inactivity and nutritional deficiencies may contribute to higher sarcopenia risk in the Indian population.

### 4.8. Correlation of HbA1c with Frailty and Sarcopenia

The study demonstrated no significant correlation between HbA1c and frailty or sarcopenia. These findings are in contrast with Alfaro-Alvarado et al. (2023), who observed that poor glycemic control (HbA1c ≥ 7.5%) was significantly associated with sarcopenia [18]. However, our findings are supported by Mange AS et al. (2021) [15], who suggested that both low HbA1c and high HbA1c levels could lead to frailty. The discrepancy may be due to differences in age, sarcopenia definitions, and population metabolic profiles. Previous literature suggests that frailty may be influenced not only by hyperglycemia but also by hypoglycemia and glycemic variability [10].

However, a very strong correlation was observed between EFS and SARC-F scores (rho = 0.856, *p* < 0.001), highlighting the close interlink between frailty and risk of sarcopenia in elderly diabetics, consistent with previous literature. The strong association between frailty and risk of sarcopenia observed in this study is consistent with the conceptual framework proposed by EWGSOP2, where muscle decline underlies physical frailty [11].

The strong correlation observed between EFS and SARC-F scores may be partially attributable to overlap in domains assessing physical function and mobility. As both tools include similar constructs such as walking ability, transfers, and stair climbing, the observed association may reflect shared measurement components in addition to an underlying biological relationship between frailty and sarcopenia.

### 4.9. Correlation of BMI with HbA1c, Frailty, and Risk of Sarcopenia

Analysis across BMI categories revealed a statistically significant difference in HbA1c levels (*p* = 0.028), with the highest mean HbA1c in Obese Class I (8.4 ± 1.98%). This supports the association of higher BMI with poor glycemic control, consistent with Alfaro-Alvarado et al., who reported that poor glycemic control is significantly linked to low muscle bulk and sarcopenia [18].

However, there was no significant difference in EFS or SARC-F scores across BMI groups. This observation may be explained by the concept of “sarcopenic obesity,” where increased fat mass coexists with reduced muscle mass, thereby masking functional decline where BMI is used as a sole indicator. Additionally, some studies, including Rehman et al. [17] found that obesity may not be a strong predictor of frailty or sarcopenia, and may even exert a paradoxical protective effect in some subgroups.

### 4.10. Limitations of the Study

The strong correlation between frailty and risk of sarcopenia (rho = 0.856, *p* < 0.001) may reflect a shared underlying pathophysiological pathway, likely mediated by chronic inflammation, hormonal dysregulation, and reduced physical activity. This reinforces the concept of “physical frailty phenotype,” where sarcopenia forms the biological substrate of frailty. Use of screening tools (SARC-F) instead of objective muscle mass measurements may have influenced prevalence estimates.

Interestingly, the absence of association with HbA1c suggests that glycemic variability or long-term metabolic burden, rather than a single HbA1c measurement, may play a more important role.

A pragmatic screening approach in clinical practice could involve initial assessment using SARC-F and EFS during routine diabetes visits in elderly patients, followed by targeted interventions in those identified as at risk.

The use of convenience sampling in a tertiary care setting may introduce selection bias, as such patients often have more advanced disease and a higher comorbidity burden compared to community-dwelling populations. Therefore, the findings of this study may not be fully generalizable to the broader elderly diabetic population. Future studies employing community-based sampling and larger, more representative populations are required to validate these findings.

Given the cross-sectional nature of this study, the observed associations should not be interpreted as causal relationships, but as a correlation. Furthermore, the relationship between glycemic control and frailty may be bidirectional, as frailty may influence metabolic control, while poor glycemic control may also contribute to functional decline. Longitudinal studies are required to better understand the temporal and causal relationships between diabetes control, sarcopenia progression, and frailty.

The assessment of glycemic control in this study was based on a single HbA1c measurement, which may not adequately reflect long-term glycemic exposure or variability. Factors such as duration of diabetes, glycemic fluctuations, hypoglycemic episodes, treatment modalities, and nutritional status were not comprehensively analyzed and may have influenced the observed relationships.

### 4.11. Clinical Implications

The findings of this study have important implications for routine clinical practice in elderly patients with type 2 diabetes mellitus. Given the high prevalence of frailty (35%) and sarcopenia risk (36.9%), routine screening using simple tools such as the Edmonton Frailty Scale (EFS) and SARC-F questionnaire may be incorporated into outpatient and inpatient settings.

The strong correlation between frailty and sarcopenia suggests that identification of one condition should prompt evaluation for the other. As these tools are quick, inexpensive, and do not require specialized equipment, they are particularly suitable for resource-limited settings.

Importantly, the lack of association with HbA1c highlights that reliance solely on glycemic parameters may fail to identify functionally vulnerable patients. Therefore, comprehensive geriatric assessment, including functional status evaluation, should be integrated into diabetes care.

Early identification of vulnerable individuals offers an opportunity for intervention through lifestyle modification, nutritional optimization, resistance exercise, and medication review, potentially preventing progression to overt frailty and disability.

## 5. Conclusions

In the present study, frailty or vulnerable was observed in 35% of the study population, while 36.9% of participants were identified to be at risk for sarcopenia. 20.6% were pre-frail, indicating a substantially higher proportion of the vulnerable group in the study population. Among the clinical presentations, fatigue emerged as the most common symptom, reported by 10.6% of the individuals, followed by polyuria and polydipsia, which were present in 4.4% of the participants. Cardiovascular disease was the most frequently encountered complication, affecting 28.1% of the population, followed by diabetic neuropathy in 15% and diabetic nephropathy in 9.4%. Analysis of glycemic control indicated a minimal correlation between HbA1c levels and the occurrence of frailty or risk of sarcopenia, likely because both hypoglycemia and hyperglycemia may contribute to frailty.

Frailty and sarcopenia are highly prevalent and strongly interrelated in elderly individuals with type 2 diabetes mellitus. Despite no significant association with HbA1c, these conditions represent critical dimensions of patient health that are not captured by glycemic control alone. Routine screening using simple, validated tools such as EFS and SARC-F may facilitate early identification and enable timely interventions. Future longitudinal studies are required to clarify causal relationships and optimize management strategies.

Routine screening for frailty and sarcopenia risk using simple tools like EFS and SARC-F should be considered in elderly diabetic care to improve early identification and guide holistic management and prevent grave consequences.

## Figures and Tables

**Table 1:** Baseline characteristics of the study group (including demographic profile, clinical features, treatment, SARC F, EFS).

Variables	Study Group
**Age Distribution**	**Frequency (%), n = 160**
60–70	89 (55.6)
71–80	61 (38.1)
81–90	10 (6.3)
Mean Age	69.98 ± 5.86
**Gender**	
Male	101 (63.1)
Female	59 (36.9)
**Body Mass Index**	
Normal	33 (20.6)
Overweight	54 (33.8)
Obese Class I	66 (41.3)
Obese Class II	7 (4.4)
**Clinical Presentation**	
Fatigue	17 (10.6)
Fever	4 (2.5)
Polyuria/polydipsia	7 (4.4)
Nausea/Vomiting	5 (3.1)
Burning Micturition	2 (1.3)
Giddiness	5 (3.1)
Headache	3 (1.9)
Breathlessness	2 (1.3)
**Disease Complication**	
Nephropathy	15 (9.4)
Neuropathy	24 (15)
Retinopathy	11 (6.9)
Peripheral Artery Disease	6 (3.8)
Cardiovascular Disease	45 (28.1)
**Comorbidity**	
Chronic Kidney Disease	15 (9.4)
Hypertension	125 (78.1)
Hypothyroidism	28 (17.5)
Ischemic Heart Disease	31 (19.4)
Dyslipidemia	32 (20)
**Treatment**	
OHA	127 (79.4)
Insulin	5 (3.1)
OHA + Insulin	28 (17.5)
Drug Compliance	154 (96.3)
**EFS severity**	
Not frail	104 (65)
vulnerable	33 (20.6)
mild frailty	15 (9.4)
moderate frailty	7 (4.4)
severe frailty	1 (0.6)
**Sarcopenia risk**	
absent	101 (63.1)
present	59 (36.9)

**Table 2:** Correlation between EFS, SARC F and glycemic control.

Correlated Variables	Correlation Coefficient	*p*-Value
EFS and HbA1c	0.087	0.276
SARC F and HbA1C	0.065	0.411
EFS and SARC F	0.856	<0.001

## Data Availability

The datasets used and/or analyzed during the current study are available from the corresponding author upon request.

## Abbreviations

The following abbreviations are used in this manuscript:

T2DM	Type 2 diabetes mellitus
EFS	Edmonton Frailty Scale
WHO	World Health Organization
BMI	Body mass index
